# Corneal Endothelial Expansion Promoted by Human Bone Marrow Mesenchymal Stem Cell-Derived Conditioned Medium

**DOI:** 10.1371/journal.pone.0069009

**Published:** 2013-07-23

**Authors:** Makiko Nakahara, Naoki Okumura, EunDuck P. Kay, Michio Hagiya, Kiwamu Imagawa, Yuuki Hosoda, Shigeru Kinoshita, Noriko Koizumi

**Affiliations:** 1 Department of Biomedical Engineering, Faculty of Life and Medical Sciences, Doshisha University, Kyotanabe, Japan; 2 Department of Ophthalmology, Kyoto Prefectural University of Medicine, Kyoto, Japan; 3 Research Division, JCR Pharmaceuticals Co., Ltd., Kobe, Japan; University of Sao Paulo – USP, Brazil

## Abstract

Healthy corneal endothelium is essential for maintaining corneal clarity, as the damage of corneal endothelial cells and loss of cell count causes severe visual impairment. Corneal transplantation is currently the only therapy for severe corneal disorders. The greatly limited proliferative ability of human corneal endothelial cells (HCECs), even *in vitro*, has challenged researchers to establish efficient techniques for the cultivating HCECs, a pivotal issue for clinical applications. The aim of this study was to evaluate conditioned medium (CM) obtained from human bone marrow-derived mesenchymal stem cells (MSCs) (MSC-CM) for use as a consistent expansion protocol of HCECs. When HCECs were maintained in the presence of MSC-CM, cell morphology assumed a hexagonal shape similar to corneal endothelial cells *in vivo*, as opposed to the irregular cell shape observed in control cultures in the absence of MSC-CM. They also maintained the functional protein phenotypes; ZO-1 and Na^+^/K^+^-ATPase were localized at the intercellular adherent junctions and pump proteins of corneal endothelium were accordingly expressed. In comparison to the proliferative potential observed in the control cultures, HCECs maintained in MSC-CM were found to have more than twice as many Ki67-positive cells and a greatly increased incorporation of BrdU into DNA. MSC-CM further facilitated the cell migration of HCECs. Lastly, the mechanism of cell proliferation mediated by MSC-CM was investigated, and phosphorylation of Akt and ERK1/2 was observed in HCECs after exposure to MSC-CM. The inhibitor to PI 3-kinase maintained the level of p27^Kip1^ for up to 24 hours and greatly blocked the expression of cyclin D1 and D3 during the early G1 phase, leading to the reduction of cell density. These findings indicate that MSC-CM not only stimulates the proliferation of HCECs by regulating the G1 proteins of the cell cycle but also maintains the characteristic differentiated phenotypes necessary for the endothelial functions.

## Introduction

Human corneal endothelium is known to play a critical role in maintaining corneal transparency by regulating corneal hydration [1]. The proliferative ability of human corneal endothelial cells (HCECs) is severely limited *in vivo* [2], therefore, cell loss due to the aging process or pathological conditions causes a concurrent compensatory migration of the existing cells and the enlargement of those cells to achieve a contact-inhibited monolayer. Maintenance of such a phenotype is necessary for functional integrity and corneal deturgescence [3], [4]. If the density of corneal endothelial cells (CECs) is below 500 cells/mm^2^, Na^+^/K^+^-ATPase pump and barrier functions are not compensated by residual CECs. The failure of endothelial functions is known to subsequently cause stromal and epithelial edema, as well as loss of corneal clarity and visual acuity. In addition, irreversible corneal haziness is often observed in corneal endothelial disorders such as Fuchs' corneal dystrophy, pseudophakic bullous keratopathy, or trauma-related injuries [5], [6].

The treatment of severe corneal disorders requires either full-thickness corneal transplantation or endothelial keratoplasty to restore clear vision. Recently, highly effective surgical techniques for the treatment of corneal disorders, i.e., Descemet's stripping automated endothelial keratoplasty (DSAEK) and Descemet's membrane endothelial keratoplasty (DMEK), have been developed, with these procedures being aimed at replacing penetrating keratoplasty [7]–[10]. However, the worldwide shortage of transplantable donor corneas, the continual cell damage after transplantation, and primary graft failure are issues that remain to be resolved [11], [12]. To overcome these problems, our group, as well as several other groups, have explored new treatment protocols for corneal endothelial dysfunctions through the use of tissue engineering techniques [13]–[17]. Among researchers worldwide, the common goal is to establish optimum experimental protocols for the *in vitro* expansion of HCECs for clinical application.

HCECs are arrested at the G1 phase of the cell cycle [2], [18], and this characteristic property of HCECs indicates that HCECs have the potential to proliferate in response to growth stimulation factors. Recently, we reported that Y-27632, a specific inhibitor of the Rho-associated coiled-coil forming kinases (ROCKs), promoted the adhesion and proliferation of monkey CECs [17], [19]. It has also been reported that FGF-2 stimulates the cell proliferation of HCECs through degradation of p27^Kip1^ (p27) [20]. The findings from these studies confirm that the proliferative potential of HCECs can be resumed and that such cells can be cultivated for clinical transplantation into the eye, thus replacing the endothelial keratoplasty. Although a variety of methods to expand HCECs *in vitro* have been explored, protocols for the expansion of HCECs for tissue engineering therapy have yet to be established [21], [22]. We recently reported that the use of conditioned medium (CM) obtained from NIH-3T3 (NIH-3T3-CM) resulted in efficient cultures of HCECs [23]. However, the use of NIH-3T3-CM faces the obstacle that CM derived from mouse cells contains a xenoantigen for human cells [24], [25]. To overcome this difficult obstacle, CM obtained from human bone marrow (BM)-derived mesenchymal stem cells (MCSs) (MSC-CM) was investigated in this present study, as BM-derived stem cells reportedly promote tissue repair by the secretion of cytokines and growth factors that enhance regeneration of injured cells, thus stimulating the proliferation and differentiation of endogenous stem-like progenitors found in most tissues [26]–[28].

In the present study, we provide evidence suggesting that CM obtained from BM-MSCs stimulates cell proliferation and motility of HCECs, while maintaining the contact-inhibited monolayer with functional adherent junctions and pump functions. Our findings show that the proliferative action of MSC-CM is facilitated via the downregulation of p27 and the upregulation of cyclin D through phosphatidylinositol 3-kinase (PI 3-kinase) and extracellular signal-regulated kinase 1/2 (ERK1/2) pathways. These results indicate that MSC-CM provides a feasible means by which to expand proliferative and functional HCECs for use as a subsequent clinical intervention for corneal endothelial dysfunction.

## Materials and Methods

### Ethics statement

The human tissue used in this study was handled in accordance with the tenets set forth in the Declaration of Helsinki. Informed written consent was obtained from the next of kin of all deceased donors in regard to eye donation for research. Human donor corneas were obtained from SightLife™ (http://www.sightlife.org/, Seattle, WA). All tissue was recovered under the tenants of the Uniform Anatomical Gift Act (UAGA) of the particular state in which the donor consent was obtained and the tissue was recovered.

### Cell cultures

All human corneas had been stored at 4°C in storage medium (Optisol; Chiron Vision, Irvine, CA) for less than 14 days prior to the use of the associated HCECs in the culture. Donor age ranged from 51 to 68 years. The culture medium was prepared according to published protocols, but with some modifications [23], [29]. The Descemet's membrane/corneal endothelium complex was stripped and digested with 1 mg/mL collagenase A (Roche Applied Science, Penzberg, Germany) at 37°C for 2 hours, followed by washing with OptiMEM-I (Life Technologies, Carlsbad, CA). HCECs obtained from the individual donor corneas were resuspended in basal growth medium (OptiMEM-I, 8% fetal bovine serum (FBS), 5 ng/mL epidermal growth factor (EGF), 20 µg/mL ascorbic acid (Sigma-Aldrich, St. Louis, MO), 200 mg/L calcium chloride, 0.08% chondroitin sulfate (Sigma-Aldrich), and 50 µg/mL gentamicin (Life Technologies)) and plated into 2 wells of a 12-well plate coated with FNC Coating Mix® (Athena Environmental Sciences, Inc., Baltimore, MD). The HCECs were maintained in a humidified atmosphere at 37°C in 5% CO_2_, and the culture medium was replaced with fresh media every 2 days. When the cells reached confluency in 14 to 28 days, they were rinsed in Ca^2+^ and Mg^2+^-free phosphate buffered saline (PBS), trypsinized with 0.05% Trypsin-EDTA (Life Technologies) for 5 minutes at 37°C, and passaged at a 1∶2 ratio. U0126 (10 µM; Wako Pure Chemical Industries, Ltd., Osaka, Japan) and LY294002 (10 µM; Wako Pure Chemical Industries, Ltd., Osaka, Japan) were used to inhibit MEK and PI 3-kinase, respectively.

### Preparation of NIH-3T3-CM

Inactivation of the 3T3 fibroblasts was performed as described previously [30], [31]. Briefly, confluent 3T3 fibroblasts were incubated with 4 µg/mL mitomycin C (MMC) (Kyowa Hakkko Kirin Co., Ltd., Tokyo, Japan) for 2 hours, and then seeded onto plastic dishes at a cell density of 2 × 10^4^ cells/cm^2^. Next, the attached cells were washed 3 times with PBS, and the medium was replaced with basal culture medium containing OptiMEM-I, 8% FBS, 5 ng/mL EGF, 20 µg/mL ascorbic acid, 200 mg/L calcium chloride, 0.08% chondroitin sulfate, and 50 µg/mL of gentamicin. The NIH-3T3 was maintained for an additional 24 hours. The medium was collected and centrifuged at 2000 xg for 10 minutes, and the supernatant was filtered through a 0.22- µm filtration unit (EMD Millipore Corporation, Billerica, MA) and used as NIH-3T3-CM.

### Preparation of MSC-CM

BM-MSCs were obtained from JCR Pharmaceuticals Co., Ltd. (Kobe, Japan). BM-MSCs passaged 3 times were used for the experiments. The BM-MSCs were plated at a cell density of 1.3 × 10^4^ cells/cm^2^ and cultured in DMEM supplemented with 10% FBS, 100 U/mL penicillin, and 100 µg/mL streptomycin, and were then maintained for 1 day. The attached cells were washed 3 times with PBS, and the medium was replaced with basal growth medium. The BM-MSCs were then maintained for an additional 24 hours. The medium was collected and centrifuged at 2000 xg for 10 minutes, and the supernatant was filtered through a 0.22- µm filtration unit (EMD Millipore Corporation) and used as MSC-CM.

### Total RNA extraction and reverse transcription polymerase chain reaction (RT-PCR)

HCECs after 5 passages were seeded at a cell density of 1.6 × 10^4^ cells/cm^2^ and maintained for 1 day, and the medium was replaced with either MSC-CM or NIH-3T3-CM. The cultures were maintained for 8 days. Total RNA was isolated by use of the RNeasy Mini kit (Qiagen, Hilden, Germany) according to manufacturer's protocol. The quality of the RNA preparations was measured by use of the NanoDrop® (Thermo Fisher Scientific Inc., Waltham, MA) spectrophotometer. First-strand cDNA was synthesized with 1 µg of total RNA by use of the ReverTra Ace® (Toyobo Corporation, Osaka, Japan) reverse transcriptase kit. The cDNA samples were subjected to PCR with specific primers as listed in Table 1; genes involved in the transport of the corneal endothelium were analyzed in comparison with glyceraldehyde 3-phosphate dehydrogenase (GAPDH) as an internal control. PCR reactions were then performed with Extaq DNA polymerase (Takara Bio Inc., Otsu, Japan) as follows: denaturation at 94°C for 30 seconds, 33 cycles of annealing at 54°C for 30 seconds, and elongation at 72°C for 30 seconds. The PCR products were separated by electrophoresis on 1.5% agarose gels, followed by ethidium bromide staining and detection under ultraviolet illumination.

**Table 1:** Oligonucleotide sequences for RT-PCR.

Gene	Sense primer	Anti-sense primer	Size (bp)
*keratin 12*	5′-GGCCTACATGAAGAAGAACCAC-3′	5′-CTCGATCTCCAGGTTCTGAAAG-3′	295
*CLCN3*	5′-GAGTTTTGCCTTTCTTGCAGTT-3′	5′-GAAAAGATATTTCCGCAGCAAC-3′	203
*VDAC3*	5′-ATAAGTTGGCTGAAGGGTTGAA-3′	5′-TTCTGTGACAGTTTGGATTTGG-3′	235
*SLC4A4*	5′-GCTTGCAGATTACTACCCCATC-3′	5′-TTGAACACTCCTTCTTCGACAA-3′	209
*p-120*	5′-AGGATCCAGCAAACGATACAGT-3′	5′-AGGTCAGCTATGGCAGAAAGAG-3′	244
*ZO-1*	5′-TTCTGAGGCCTGTAACCATTTT-3′	5′-AATTGGATACCACTGGGCATAG-3′	245
*Na^+^/K^+^-ATPase*	5′-ACGGCAGTGATCTAAAGGACAT-3′	5′-GAAGAATCATGTCAGCAGCTTG-3′	255
*GAPDH*	5′-GAGATCCCTCCAAAATCAAGTG-3′	5′-GAGTCCTTCCACGATACCAAAG-3′	245

### Cell proliferation assay

HCECs were cultured at the density of 5000 cells/well in a 96-well plate in the presence or absence of CM derived from NIH-3T3 or BM-MSC. DNA synthesis was detected as incorporation of 5-bromo-2′-deoxyuridine (BrdU) into DNA by use of the Cell Proliferation Biotrak ELISA system, version 2 (GE Healthcare Life Sciences, Buckinghamshire, UK) according to the manufacturer's instructions. Briefly, HCECs were incubated with 10 µM BrdU for 24 hours at 37°C and 5% CO_2_ in a humidified atmosphere. Cultured cells were incubated with fixation solution for 2 hours and incubated with 100 µl of monoclonal antibody against BrdU for 30 minutes. The BrdU absorbance was measured directly using a spectrophotometric microplate reader at a test wavelength of 450 nm.

### Immunofluorescent staining

Cultured HCECs on a 48-well cell culture plate were fixed in 4% paraformaldehyde for 10 minutes at room temperature and then incubated for 30 minutes with 1% bovine serum albumin (BSA). Immunocytochemical analyses of ZO-1 (Zymed Laboratories, South San Francisco, CA) and Na^+^/K^+^-ATPase (Upstate Biotec, Lake Placid, NY) were respectively performed with a 1∶200 dilution of ZO-1 polyclonal antibody and a 1∶200 dilution of Na^+^/K^+^-ATPase monoclonal antibody. Either Alexa Fluor® 488-conjugated goat anti-mouse (Life Technologies) or Alexa Fluor® 594-conjugated goat anti-rabbit IgG (Life Technologies) was used for the secondary antibody with a 1∶1000 dilution. Nuclei were stained with DAPI (Vector Laboratories, Burlingame, CA).The cells were then examined by fluorescence microscopy (BZ-9000; Keyence, Osaka, Japan).

### Scratch-induced directional migration assay

HCECs were cultured in 6-well plates in basal growth media. When the cells reached confluence, they were maintained in either control basal growth medium, NIH-3T3-CM, or MSC-CM for an additional 7 days. Scrape-wounding of the cells was performed using a plastic pipette tip. Following scraping, the medium containing detached cells was removed and replaced with basal growth medium, NIH-3T3-CM, or MSC-CM; cells were further maintained for 20 hours until the monolayer was restored. Cell migration and the recovery to a cell monolayer were determined by phase contrast microscopy. The width of the wound area was measured using ImageJ software (U.S. National Institutes of Health, Bethesda, MD), and three fields from each well were measured.

### Western blot analysis

The HCECs were washed with ice-cold PBS and then lysed with ice-cold RIPA buffer (Bio-Rad Laboratories, Hercules, CA) containing Phosphatase Inhibitor Cocktail 2 (Sigma-Aldrich) and Protease Inhibitor Cocktail (Nacalai Tesque, Kyoto, Japan). The lysates were centrifuged at 15,000 rpm for 10 minutes at 4°C to sediment debris. The supernatant representing total proteins was collected and the protein concentration of the sample was assessed by use of the BCA™ Protein Assay Kit (Takara Bio). An equal amount of protein was fractionated by SDS-PAGE; proteins were transferred to PVDF membranes. The membranes were then blocked with 3% non-fat dry milk (Cell Signaling Technology, Inc., Danvers, MA) in TBS-T buffer (50 mM Tris, pH 7.5, 150 mM NaCl_2_, and 0.1% Tween20) for 1 hour at room temperature, followed by overnight incubation at 4°C with the following primary antibodies: Na^+^/K^+^-ATPase (1∶1000; Merck Millipore), ZO-1 (1∶1000; Zymed Laboratories), GAPDH (1∶3000; Abcam, Cambridge, UK), Akt1 (1∶2000; Cell Signaling Technology), phosphorylated Akt (1∶2000; Cell Signaling Technology), ERK1/2 (BD, Franklin Lakes, NJ), and phosphorylated ERK1/2 (BD). The blots were washed, and then incubated with horseradish peroxidase-conjugated secondary antibodies (1∶5000: anti-rabbit IgG, anti-mouse IgG; Cell Signaling Technology). The blots were then developed with luminal for enhanced chemiluminescence (ECL) using the ECL Advance Western Blotting Detection Kit (GE Healthcare, Piscataway, NJ), documented by LAS4000S (Fuji Film, Tokyo, Japan), and analyzed with Image Gauge (Fuji Film).

### Concentration of MSC-CM

Supernatants of MSC cultured in OptiMEM-I supplemented with gentamicin were collected after 24 hours. After centrifugation at 1000 rpm for 10 minutes to remove cell debris, cell-free supernatant was concentrated 17-fold by centrifugation at 2500 xg for 3 hours using Ultra-PL 3 ultrafiltration units (Amicon; EMD Millipore) with a 3-kDa molecular weight cutoff.

### Statistical analysis

The statistical significance (*P*-value) in mean values of the two-sample comparison was determined with the Student's t-test. The statistical significance in the comparison of multiple sample sets was analyzed with Dunnett's multiple-comparisons test. Results were expressed as mean ± SEM.

## Results

### MSC-CM and NIH-3T3-CM maintain corneal endothelial phenotype in vitro expansion

**Figure 1:**
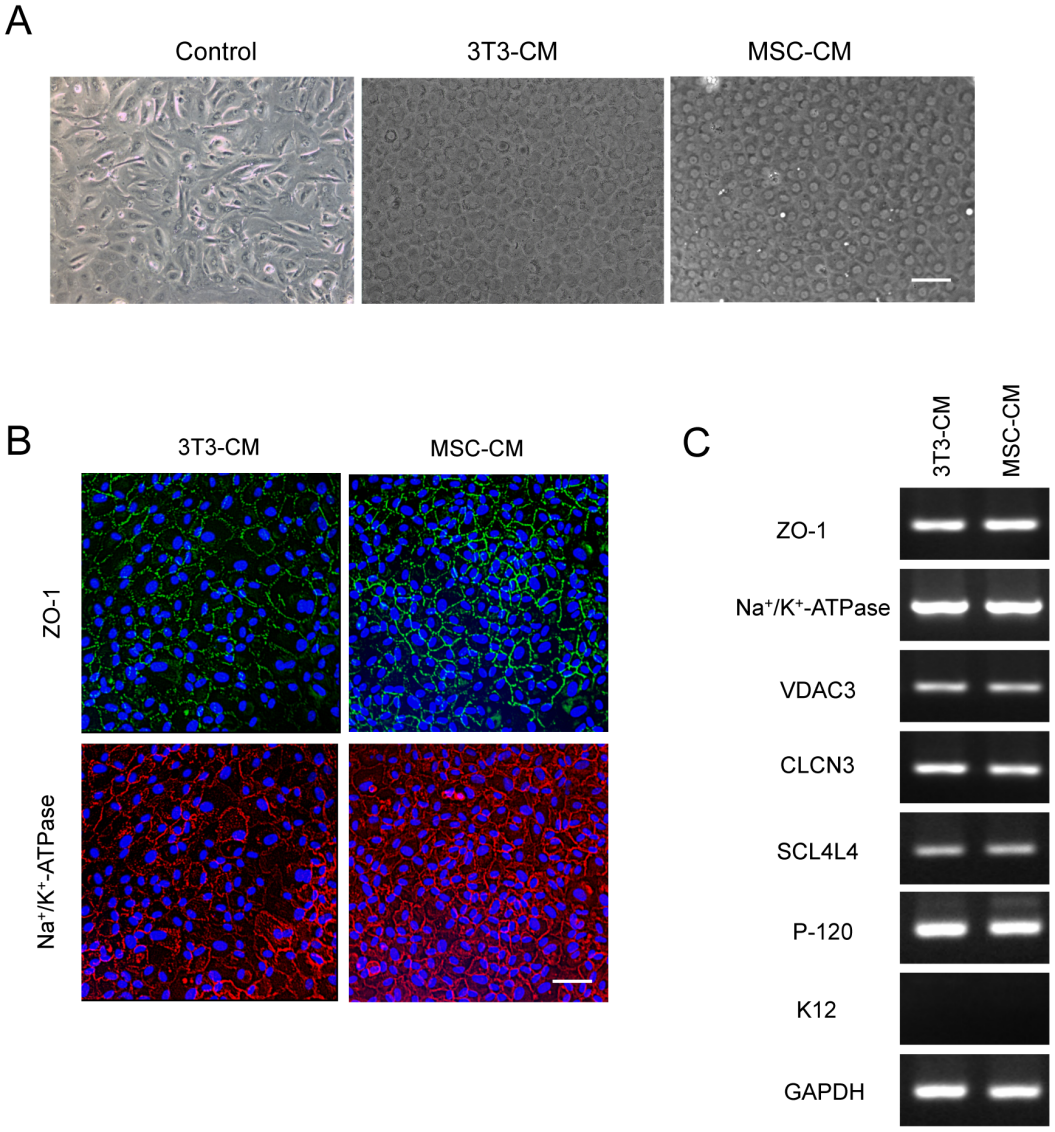
MSC-CM and NIH3T3-CM maintain corneal endothelial phenotype *in vitro* expansion. (A) Effect of MSC-CM on morphology of primary cultures of HCECs. Representative phase-contrast images of primary culture from different CMs. Cultured HCECs were maintained in basal growth medium, MSC-CM, or NIH3T3-CM for 30 days. Scale bar: 200 µm. (B) HCECs cultured in either MSC-CM or NIH3T3-CM for 14 days expressed ZO-1 and Na^+^/K^+^-ATPase. The pictures are representative of 2 independent experiments. (C) Expression of genes involved in the active transmembrane transporter activity in HCECs cultured with both NIH3T3-CM and MSC-CM was assessed by RT-PCR. The experiments were performed in duplicate.

Current isolation and cultivation methods to establish HCECs *in vitro* face an unexpected obstacle due to spontaneous morphological fibroblastic change and severely limited proliferative ability. Therefore, we tested the CM obtained from human BM-MSCs in this study. HCECs were maintained in basal growth medium, NIH-3T3-CM, or MSC-CM for 30 days. The control cells maintained in basal growth medium showed loss of the characteristic polygonal cell morphology, whereas HCECs maintained in either CM demonstrated a contact-inhibited monolayer of hexagonal cells (Figure 1A). Immunostaining of ZO-1 and Na^+^/K^+^-ATPase was clearly outlined at the intercellular adherent junction in HCECs maintained with either MSC-CM or NIH-3T3-CM (Figure 1B), similar to the previous findings [32]. Expression of genes involved in the active transmembrane transporter activity was assessed by RT-PCR (Figure 1C). The transcripts of ZO-1, Na^+^/K^+^-ATPase, voltage dependent anion channel3 (VDAC3), chloride channel protein 3 (CLCN3), sodium bicarbonate co-transporter member4 (SLC4A4), and p-120 were expressed in HCECs, while keratin 12 (K12) was not expressed.

### Effect of MSC-CM on the proliferation of HCECs

**Figure 2:**
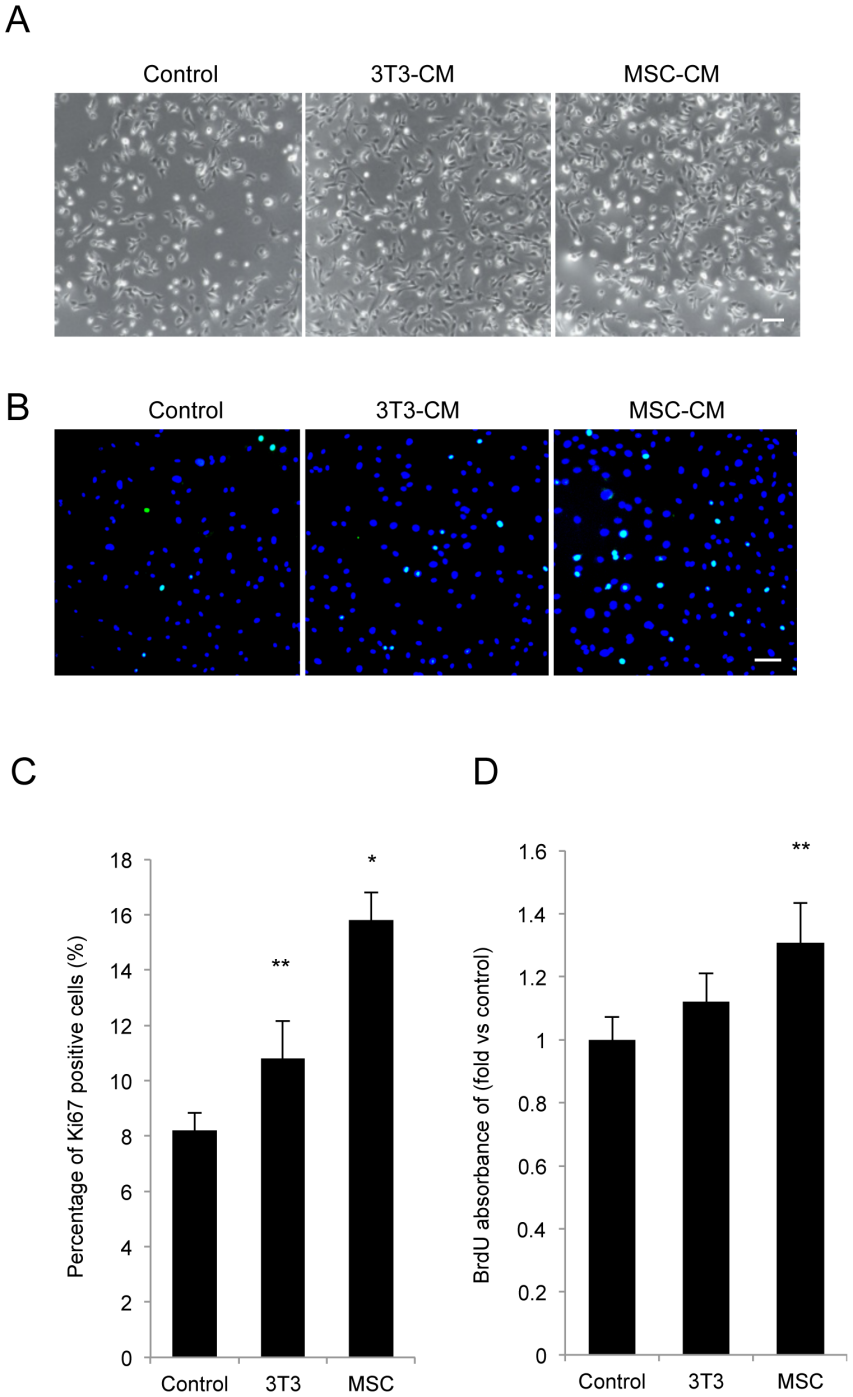
MSC-CM enhances the proliferation of HCECs. (A) Phase-contrast images of HCECs cultured with MCS-CM. HCECs were seeded and cultured with MSC-CM, NIH3T3-CM, or basal growth medium (control) for 5 days. Scale bar: 200 µm. (B+C) To test proliferative potential, HCECs maintained for 5 days under the experimental conditions were immunostained with the cell-cycle-progression population marker Ki67, and the percentages of Ki-67 positive cells were then evaluated. The experiment was performed in duplicate. Scale bar: 200 µm. (D) HCECs were cultured in basal growth medium (control), NIH3T3-CM, or MSC-CM. Proliferation of HCECs was evaluated by BrdU incorporation assay after 5 days of incubation. The experiment was performed in triplicate. * *p*<0.01, ** *p*<0.05.

HCECs were cultured in basal growth medium, MSC-CM, or NIH-3T3-CM (Figure 2A), and the proliferative potential was then assessed using two respective methods: Ki67 staining and BrdU incorporation into the newly synthesized DNA. HCECs maintained for 5 days under the experimental conditions were immunostained with the cell cycle progression population marker Ki67 (Figure 2B). The control cells showed 8.2% Ki67-positive cells, whereas HCECs treated with MSC-CM showed 15.8% Ki67-positive cells (Figure 2C). When incorporation of BrdU into the newly synthesized DNA was measured, HCECs maintained in MSC-CM showed a much higher incorporation of BrdU into DNA than did the control cells (Figure 2D). Of interest, HCECs maintained in NIH-3T3-CM demonstrated lower proliferative potential when compared to HCECs maintained in MSC-CM

### Effect of MSC-CM on the wound closure of HCECs

**Figure 3:**
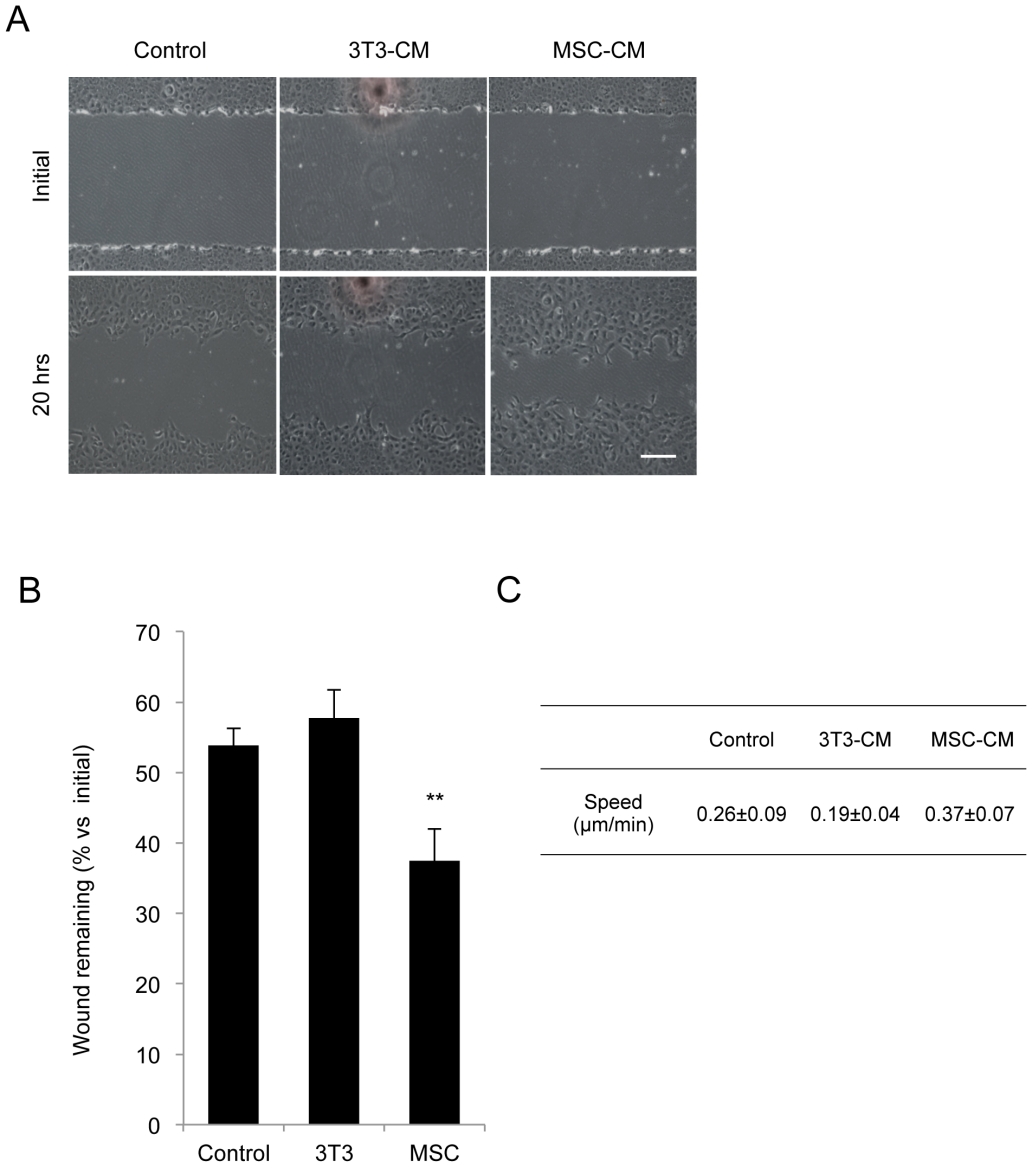
MSC-CM promotes cell motility in an *in vitro* wound model. (A+B) HCECs were cultured with basal growth medium (control), NIH3T3-CM, or MSC-CM for 40 days, and the monolayer cells were then wounded by scratching. After 20 hours, the remaining wound area was quantified by Image J software. ** *p*<0.05. Scale bar: 200 µm. (C) The speed of cell motility was measured from the image. The experiments were performed in triplicate.

Scratch-induced directional migration assay was employed to compare the wound closure in HCECs maintained in MSC-CM to those of control cells and the NIH-3T3-CM-treated cells (Figure 3A, B). The wound was introduced to the confluent cultures and wound closure was measured 20 hours after the initial wounding. Cells maintained in MSC-CM demonstrated the fastest healing rate; 63% of the initial wound area was covered with cells. On the other hand, both the control cells and the cells maintained in NIH-3T3-CM showed that much less area of the initial wound was recovered by cells. When wound healing over a 20-hour period was converted into the migration rate of HCECs, the MSC-CM-treated HCECs demonstrated 0.37 µm/min, the control cells showed 0.26 µm/min, and the NIH-3T3-CM-treated cells showed 0.19 µm/min (Figure 3C), similar to the earlier findings [33].

### Effect of MSC-CM-derived factors on CEC proliferation

**Figure 4:**
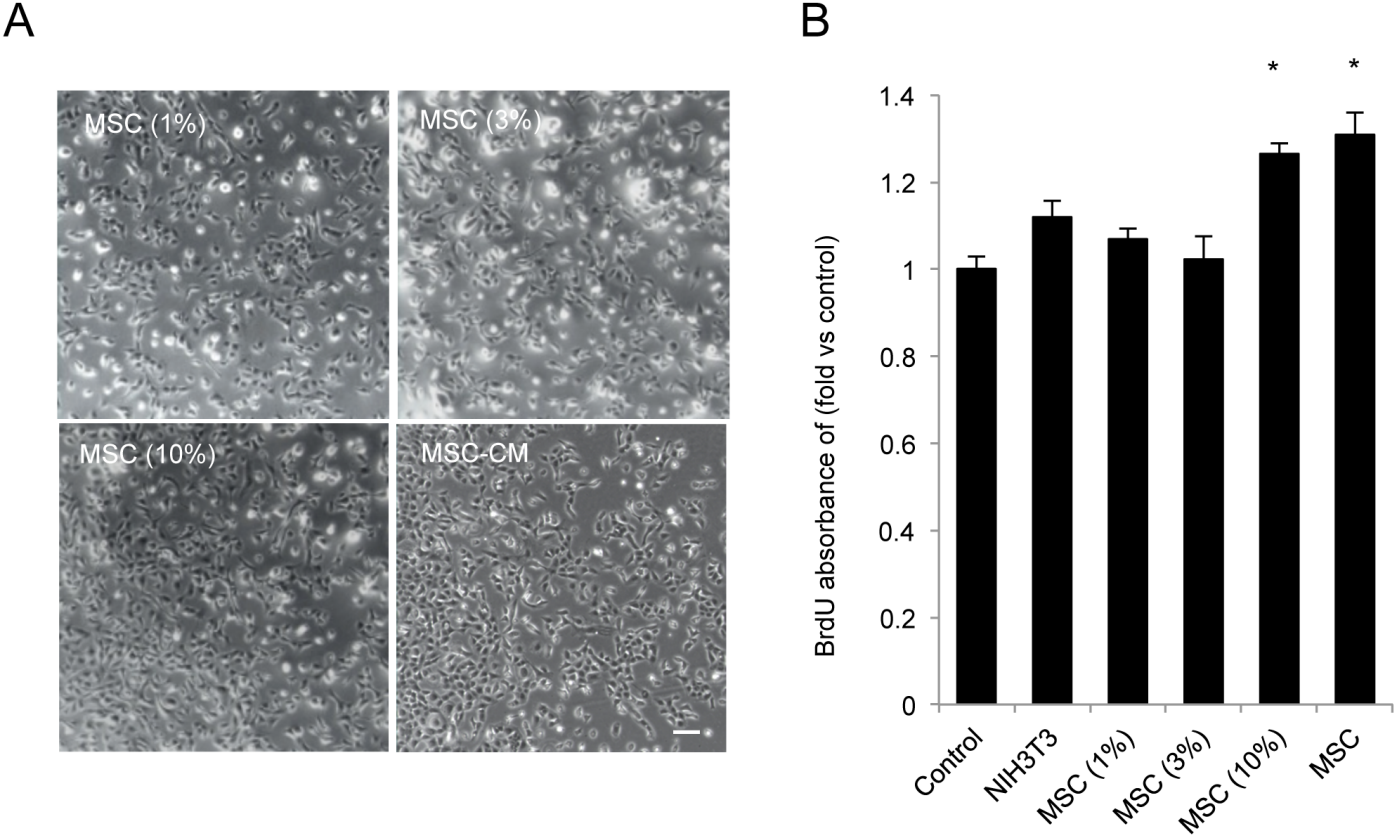
MSC-CM-derived factors enhance HCEC proliferation. (A) HCECs were maintained with basal growth medium supplemented with 1, 3, or 10% of concentrated MSC-CM or full strength MSC-CM. (B) The effect of soluble factors from MSC-CM on the proliferation of HCECs was evaluated by BrdU incorporation assay after 4 days of incubation. * *p*<0.01. The experiments were performed in duplicate. Scale bar: 200 µm.

The fact that the full strength of MSC-CM exerted proliferative activity led us to examine whether or not there is a dilution-dependent activity of MSC-CM on the proliferation of HCECs. To test the dilution effect, MSC-CM was concentrated and added to basal growth medium at the final concentration of 1%, 3%, or 10%. Then, the proliferative activity of the concentrated MSC-CM was compared to that of the full-strength CM (Figure 4A, B). Cells maintained in 10%-strength MSC-CM showed BrdU incorporation into DNA similar to the level achieved with the full-strength CM. On the other hand, MSC-CM at the strength of 1% and 3% produced no proliferative activity. These findings indicated that the soluble factors derived from MSC promote proliferation of HCECs, and also that the effect is dose-dependent.

### Involvement of PI 3-kinase and ERK1/2 in the proliferation of HCECs in response to MSC-CM stimulation

**Figure 5:**
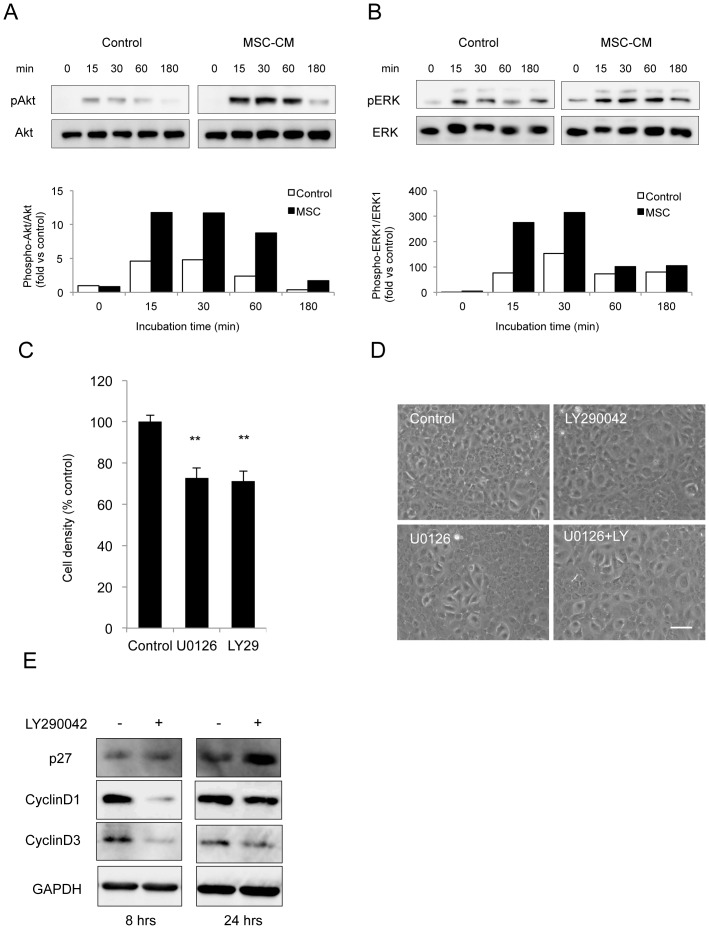
Involvement of PI 3-kinase and ERK1/2 in the proliferation of HCECs in response to MSC-CM stimulation. (A+B) HCECs were cultured without serum for 24 hours followed by treatment with MSC-CM for 15, 30, 60, or 180 minutes. Phosphorylation of Akt and ERK1/2 was evaluated by Western blot analysis. The experiments were performed in duplicate. (C+D) HCECs were cultured in the presence of the PI 3-kinase inhibitor (LY294002) or MEK inhibitor (U0126). Cell density was evaluated via the use of phase contrast microscopy. The experiments were performed in duplicate. Scale bar: 200 µm. (E) HCECs were cultured without serum for 24 hours, and then treated with MSC-CM in the absence or presence of LY294002. Expression of p27, cyclin D1, and cyclin D3 was evaluated by Western blot analysis, both at the early G1 phase (8 hours) and the late G1 phase (24 hours).

It has been known that CECs, regardless of the species, utilize PI 3-kinase and ERK1/2 pathways for cell proliferation mediated by FGF-2 [20], [34]. Therefore, we tested whether or not MSC-CM activated the PI 3-kinase and ERK1/2 pathways. When serum-starved cells were treated with MSC-CM for 15, 30, 60, or 180 minutes, phosphorylation of Akt was greatly induced from 15 minutes following treatment of the cells with CM. Such enhancement on the phosphorylation of Akt sustained for 60 minutes, after which the phosphorylation of Akt was greatly reduced (Figure 5A). The control cells showed faint levels of phosphorylated Akt. Phosphorylation of ERK1/2 was also enhanced 15 minutes following treating the cells with MSC-CM, and such phosphorylation attenuated up to 180 minutes (Figure 5B). To test whether or not the cell proliferation was induced by either PI 3-kinase or MEK, cell density was measured in the presence of the respective inhibitors to PI 3-kinase and ERK1/2; both LY294002 (PI 3-kinase inhibitor) and U0126 (MEK inhibitor) were found to block cell proliferation (Figure 5C). The cells treated with either inhibitor showed an enlarged cell shape due to the lesser cell numbers (Figure 5D). Cell proliferation observed in CECs was linked to the degradation of p27, the potent inhibitor of the G1 phase of the cell cycle [20], [34]. Therefore, we examined the amount of p27 in the absence or presence of LY294002 at the early G1 phase (8 hours) or the late G1 phase (24 hours). p27 appeared to be maintained at a low level regardless of the G1 stage in the presence of MSC-CM (Figure 5E), whereas p27 level was greatly increased in the presence of LY294002 during the late G1 phase of the cell cycle. On the other hand, cyclin D1 and cyclin D3 expressed in the presence of MSC-CM were greatly reduced by the action of LY294002 during the early G1 phase (8 hours) of the cell cycle (Figure 5E). These findings indicated that MSC-CM may employ PI 3-kinase signaling to regulate cell cycle progression through the action on p27 and cyclin D.

## Discussion

Human corneal endothelium is a physiologically important monolayer of the cornea, as the simple but crucial role of the endothelium is to maintain cornea clarity. In order to keep the entire cornea transparent, it is essential for corneal endothelium to retain the unique contact-inhibited monolayer, through which the tissue operates active pump and barrier functions. Decompensation of the corneal endothelium resulting from various causes ultimately leads to its inability to efficiently pump fluid out of the stroma, thus leading to corneal edema, loss of visual acuity, and cornea-related blindness. The function of the endothelium is compromised if the cell density falls below a critical threshold of 500 cells/mm^2^. In the United States, over 40,000 corneal transplantations were performed in 2011 [35]. Since corneal endothelial dysfunction is the major indication for performing corneal transplantations, endothelial keratoplasty represented over 40% of all corneal grafts performed in both 2009 and 2010 [36].

Various methods have been attempted to treat endothelial dysfunction, and in the most recent years, DSAEK and DMEK have been extensively employed [7]–[9], [37]. However, these relatively new procedures still face some obstacles, such as the worldwide shortage of transplantable donor corneas, continuing cell loss after transplantation, technical difficulty, and primary graft failure [11], [12]. In order to address the obstacle produced by the worldwide shortage of donor corneas, the idea of one donor cornea treating one patient has been challenged, and the concept of using one donor cornea for treating multiple patients has been widely accepted. Such a timely goal prompts researchers to establish optimum technologies for isolation and cultivation of HCECs, with the cultivated cells then being used for transplantation as a new clinical intervention for corneal endothelial dysfunction.

To achieve such a goal, our group demonstrated that transplantation of cultivated CECs in combination with a ROCK inhibitor enables the injection of the cells into the anterior chamber to regenerate corneal endothelium as a functional monolayer in an animal model [16], [17]; and this technique has the potential to be applied in the clinical setting if developed properly. However, this treatment pathway faces practical difficulties, as HCECs are arrested at the G1 phase of the cell cycle *in vivo* [2], [18] and they do not readily proliferate *in vitro*. Worse yet, HCECs naturally exhibit massive fibroblastic change with loss of pump and barrier functions during *in vitro* cultivation. To overcome such undesired events, our group has successfully used CM obtained from NIH-3T3 fibroblasts, which maintain endothelial phenotypes [23]. Similarly, mouse ESC-CM was used to enhance cell proliferation and survival of HCECs in culture [38]. Nonetheless, the use of NIH-3T3-CM or mouse ESC-CM faces a major obstacle in that the CM of the mouse cell cultures contains a xenoantigen for human cells [24], [25]. To overcome this issue, we tested the effect of CM obtained from GMP-grade human BM-derived MSCs for application in the clinical setting.

In this present study, we demonstrated findings critical for the successful cultivation protocols of HCECs that may be used for transplantation in the clinical setting. When HCECs were maintained in the presence of MSC-CM, cell morphology assumed a hexagonal shape similar to the corneal endothelial cells *in vivo*. The HCECs also maintained the functional phenotypes; ZO-1 and Na^+^/K^+^-ATPase were localized at the intercellular adherent junctions and major pump proteins (VDAC3, CLCN3, SLC4A4, and p120) present in HCECs were accordingly expressed. MSC-CM facilitated cell motility of HCECs. Of importance, MSC-CM was found to greatly stimulate the proliferation of HCECs through the PI 3-kinase and ERK1/2 pathways by degrading p27, similar to the findings reported previously [20]. In addition to its action on p27, MSC-CM was found to upregulate the expression of cyclin D1 during the early G1 phase of the cell cycle, which is another crucial step for G1/S progression.

Taken together, our findings indicate that MSC-CM not only stimulates the proliferation of HCECs by regulating the G1 proteins of the cell cycle, but also maintains the characteristic differentiated phenotypes necessary for the endothelial functions. Such dual cellular activities (proliferation and differentiation), in opposite nature, are employed for the self-renewal of stem cells/progenitors in adult tissues. Our findings suggest that HCECs maintained in MSC-CM acquire the stem-cell-like properties, which subsequently regenerate HCECs into functional corneal endothelium. These findings are the first evidence to show that when treated with MSC-CM, HCECs retain the required proliferative potential with the capacity to be fully differentiated. Therefore, the findings of this study may provide a feasible means by which to bolster the current concerted efforts to establish functioning HCECs with high growth potential [22], [39]. Thus, a combination of tissue-engineered human corneal endothelium coupled with surgical procedures presents a possible roadmap by which to treat endothelial dysfunctions.
